# Acute Fulminant Hepatic Failure and Renal Failure Induced by Oral Amiodarone: A Case Report and Literature Review

**DOI:** 10.7759/cureus.8311

**Published:** 2020-05-27

**Authors:** Natasha Campbell, Khushboo Agarwal, Marjan Alidoost, Jeffrey A Miskoff, Mohammad Hossain

**Affiliations:** 1 Internal Medicine, Jersey Shore University Medical Center, Neptune City, USA; 2 Internal Medicine, Hackensack Meridian School of Medicine at Seton Hall University, Nutley, USA; 3 Internal Medicine - Critical Care - Sleep Medicine, Ocean Medical Center, Brick, USA; 4 Internal Medicine - Critical Care - Sleep Medicine, Jersey Shore University Medical Center, Neptune City, USA

**Keywords:** amiodarone, drug toxicity, fulminant hepatic failure

## Abstract

Amiodarone is a class III antiarrhythmic agent that inhibits adrenergic stimulation by blocking alpha and beta receptors. It prolongs action potential and refractory period in myocardial tissue. Its remarkably long half-life is associated with a myriad of adverse events. Here, we present an 85-year-old male patient who was started on amiodarone for atrial flutter. After three oral doses, he developed fulminant hepatic failure and acute renal failure, which resolved after stopping amiodarone. While fulminant hepatic failure is rare, it has been seen in less than 2% of patients. Alternative theories behind susceptibility to amiodarone-induced hepatic injury and acute kidney injury are discussed here.

## Introduction

Amiodarone was initially synthesized and tested as an antianginal agent in the 1960s and its antiarrhythmic function was discovered at a later date [1]. It has been extensively prescribed as it effectively manages both supraventricular and ventricular arrhythmias [1]. Amiodarone is categorized as a class III antiarrhythmic agent that primarily inhibits adrenergic stimulation by blocking both alpha and beta receptors. It also affects sodium, potassium, and calcium channels and prolongs the action potential and refractory period in myocardial tissue [1]. By doing so, it decreases the atrioventricular (AV) conduction and sinus node function. Amiodarone is slowly and widely distributed and is highly lipophilic. It has a remarkable volume of distribution and is metabolized by the liver via cytochrome P4502C8 (CYP2C8) and cytochrome P450 3A4 (CYP3A4) [2]. Enteral administration takes between two and 21 days to fully achieve the antiarrhythmic effect. Interestingly, a single oral dose of oral amiodarone has a mean half-life elimination of 58 days [2]. With this being said, amiodarone is associated with a high incidence of adverse events and patients must be monitored closely for any complications. Therefore, the purpose of this case is to investigate alternative hypothesis associated with amiodarone-related hepatic injuries.

## Case presentation

An 85-year-old male with a medical history pertinent for atrial flutter on coumadin and hypertension developed new onset of shortness of breath. At baseline he could ambulate without issues but was getting short of breath walking 150 feet. He also endorsed paroxysmal nocturnal dyspnea and orthopnea. His symptoms began a week before he was admitted to the hospital. He first went to this cardiologist who did an outpatient echocardiogram that showed severe systolic heart failure with an ejection fraction of 30%, a moderate mitral regurgitation, and a dilated left ventricle. He was referred to the hospital for a nuclear stress test. However, as his shortness of breath was worsening he instead went to the ED where he was found to be in atrial flutter with rapid ventricular response (RVR) with a heart rate of 130 in a 2:1 AV block. On presentation, vital signs were as follows: blood pressure 120/80 mmHg, heart rate 130 beats per minute (bpm), pulse ox 96% on two liters nasal cannula, respiratory rate 18, and temperature 99.8°F orally. It was thought that his newly diagnosed congestive heart failure (CHF) was tachycardia induced. In the ED his electrocardiogram (EKG) showed atrial flutter with RVR and no acute ST changes, and his troponins were mildly elevated, peaking at 0.07. He was admitted in the hospital and started on IV cardizem infusion per protocol and the patient was given a loading dose of oral amiodarone. On physical exam, the patient had jugular venous distension, normal sinus rhythm with systolic murmur, mild crackles bilaterally on auscultation of his lungs, a distended abdomen with dullness to percussion, and positive shifting fluid wave without palpable hepatosplenomegaly. He had trace lower extremity edema bilaterally and no changes on neurological exam. Laboratory investigation revealed (Table 1) hyponatremia of 130 mmol/L, glomerular filtration rate (GFR) >60, mildly elevated liver function tests (LFTs) with aspartate aminotransferase (AST) of 48 iU/L and normal alanine aminotransferase (ALT) of 42 iU/L. Complete blood count (CBC) was pertinent for mild leukocytosis of 13.5 K/uL and microcytic anemia with a hemoglobin of 11.8 g/dL, which was around his baseline. He was found to have coumadin coagulopathy with an INR of 7.74. Of note, brain natriuretic peptide (BNP) was 863 on admission. His coumadin was held and he was given oral 5 mg vitamin K. Chest X-ray showed bilateral pleural effusions and some vascular congestion. He was initially watched with telemonitoring on the medical floors. He received Lasix IV after which his shortness of breath resolved, and he no longer appeared to be in CHF exacerbation. On his first day after admission his LFTs were within normal limits (AST/ALT 33/40). His second day of admission, after he had received a total of three doses of 800 mg of oral amiodarone, he began to have transaminitis, with AST/ALT of 3842/1665. He was also found to have acute renal failure with a GFR of 24. INR continued to be elevated at 8.7 and he was given another dose of oral 5 mg vitamin K. At that point, the patient was started on a N-acetylcysteine drip and transferred to the medical intensive care unit (MICU) for close monitoring. At that time, his oral amiodarone was discontinued and work-up was sent to evaluate the etiology of his fulminant hepatic failure. Gastroenterology and nephrology were consulted. Per nephrology, he was given some IV hydration due to the possibility of his acute renal failure being of pre-renal etiology. Eosinophils in the urine and blood were negative. Hepatitis panel, anti-nuclear antibodies (ANA), c-peptide, anti-double-stranded DNA, and immunoglobulin G (IgG) to glomerular basement membrane were within normal limits. Total complement level was low at 35 (normal 60-144 units). LFTs continued to rise to a maximum of AST/ALT 11675/4960 iU/L (Table 1).

**Table 1:** Summary of laboratory investigations baseline, after amiodarone and follow up after stopping amiodarone. GFR: glomerular filtration rate; AST: aspartate aminotransferase; ALT: alanine transaminase; INR: international normalized ratio.

Biochemistry						
	Baseline	On admission	22 hours after amiodarone	At 36 hours	At 10-day follow-up	Reference values
Blood urea nitrogen	16	17	29	36	11	5-25 mg/dL
Creatinine	1.09	1.16	2.97	3.23	0.97	0.61-1.24 mg/dL
GFR	>60	>60	20	18	>60	70-99 mg/dL
AST	33	48	3842	11675	64	10-42 Iu/L
ALT	40	42	1665	4960	174	10-60 Iu/L
Alkaline phosphatase	55	64	129	133	98	38-126 Iu/L
Total bilirubin	1.2	0.9	3.3	5.5	3.2	0.2-1.3 mg/dL
Direct bilirubin				2.2		
INR	3.5	7.74	8.70	4.80	1.88	0.88-1.15

Within three days of discontinuing amiodarone, the patient’s liver function and renal function started to improve. Etiologies considered for the patient’s fulminant liver failure included viral hepatitis, acetaminophen toxicity, salicylate toxicity, ischemia, CHF exacerbation, Wilson’s disease, and autoimmune hepatitis. Work-up, as detailed above, for all these etiologies came back negative. Due to the timing of the patient’s fulminant liver failure, it was discussed and agreed upon by the medical, gastrointestinal, and nephrology team that the patient’s acute liver failure was most likely due to the three doses of oral amiodarone he received. His LFTs continued to downtrend and he was eventually discharged to rehab.

## Discussion

In 25%-50% of patients who have been started on amiodarone, a transient rise in LFTs has been seen; therefore, it is recommended that serum aminotransferases be followed every six months. Rarely do patients suffer symptomatic hepatitis, cirrhosis and fulminant hepatic failure; however, it has been seen in less than 2% of patients who were started on amiodarone [3]. Although the exact duration of treatment or number of doses of amiodarone associated with hepatotoxicity has not been elucidated, severe cases of amiodarone hepatotoxicity has been associated with a higher cumulative dose [4]. Acute renal failure or renal insufficiency has been reported in less than 1% of patients who were treated with amiodarone [2]. 

Our patient only received three oral doses of 800 mg of amiodarone before his LFTs rose more than 10-fold. Tsuda et al. reported two cases of amiodarone-induced reversible and irreversible hepatotoxicity [5]. The first case was a 75-year-old male on hemodialysis with a history of mild systolic heart failure and ventricular tachycardia (VT), which was being treated with amiodarone for one year. He was admitted for asymptomatic elevation of his liver enzymes. Immunological and infectious workup was negative. Liver biopsy was consistent with amiodarone-induced nonalcoholic steatohepatitis. Transaminases returned to baseline within eight months of amiodarone cessation. The second case described was a 65-year-old male with a history of tobacco abuse and cardiac sarcoidosis complicated by VT, which was treated with amiodarone for 13 years. He was admitted for systemic edema. Imaging with abdominal ultrasound and abdomen CT scan showed liver cirrhosis, massive ascites, and splenomegaly. Amiodarone was discontinued, however, the patient died of hepatic insufficiency and autopsy with electron microscopy revealed lysosomal lamellar bodies [5]. 

Robin et al. reported a rare case like ours of a 65-year-old male with coronary artery disease (CAD) status post coronary artery bypass graft (CABG), ischemic cardiomyopathy status post automatic implantable cardioverter defibrillator (AICD) who presented with AICD shocks. He was placed on continuous intravenous amiodarone infusion and was periodically hypotensive. He developed hepatotoxicity and acute renal failure. The renal failure was attributed to the hypotensive episodes that the patient suffered [6]. Our patient's blood pressure remained stable throughout his hospitalization and renal function significantly improved after oral amiodarone was discontinued. Some cases reporting renal failure associated with amiodarone infusion were found to be secondary to cardiorenal syndrome versus rhabdomyolysis [7]. Our patient had neither of these. Interestingly, intravenous amiodarone is prepared with vehicles known as polysorbate-80 or polyoxyethylene-sorbitan-20 monooleate to make a stable solution. These vehicles are used in Vitamin E infusions, which has been reported to cause hepatotoxicity and nephrotoxicity in infants [8]. However, our patient only received oral amiodarone, excluding this mechanism of nephrotoxicity.

There are alternative theories behind why some patients are more susceptible to amiodarone-induced hepatic injury. Metabolic idiosyncrasy is a term that has been used to describe these patients who have been found to produce toxic metabolites to a greater degree than others [9-10]. On the other hand, phospholipidosis is an alternative process that also causes direct hepatotoxicity by interaction between the phospholipid and amiodarone which leads to a complex that prevents breakdown of phospholipid molecules themselves, allowing the drug to stay in the patient's system for prolonged periods of time [11]. 

It is also important to consider drug interactions when starting a patient on amiodarone. Our patient was also on warfarin. Amiodarone causes coumadin coagulopathy by its direct and indirect interference with the hepatic metabolism of warfarin. Amiodarone can potentially affect thyroid function and cause thyrotoxicosis and hypothyroidism. Thyrotoxicosis potentiates warfarin, while hypothyroidism dampens its effect [12]. Hence, patients on warfarin should have a dose reduction of 25% if amiodarone is being started [13-14].

## Conclusions

Amiodarone has the potential to adversely affect multiple organ systems. It is of utmost importance to closely monitor patients with baseline and follow up EKGs, chest radiographs, thyroid function tests, renal function tests, LFTs, pulmonary function tests, and eye examinations. If LFTs start to rise as seen in our patient, amiodarone must be stopped immediately. From this case, it can be argued that patients in the hospital should have their bloodwork monitored for at least 24-48 hours after receiving the first doses of amiodarone to prevent irreversible organ damage.
